# Stem Cell Transcription Factor FoxO Controls Microbiome Resilience in *Hydra*

**DOI:** 10.3389/fmicb.2018.00629

**Published:** 2018-04-03

**Authors:** Benedikt M. Mortzfeld, Jan Taubenheim, Sebastian Fraune, Alexander V. Klimovich, Thomas C. G. Bosch

**Affiliations:** Zoological Institute, Christian-Albrechts-Universität zu Kiel, Kiel, Germany

**Keywords:** aging, microbiome, FoxO, resilience, *Hydra*

## Abstract

The aging process is considered to be the result of accumulating cellular deterioration in an individual organism over time. It can be affected by the combined influence of genetic, epigenetic, and environmental factors including life-style-associated events. In the non-senescent freshwater polyp *Hydra*, one of the classical model systems for evolutionary developmental biology and regeneration, transcription factor FoxO modulates both stem cell proliferation and innate immunity. This provides strong support for the role of FoxO as a critical rate-of-aging regulator. However, how environmental factors interact with FoxO remains unknown. Here, we find that deficiency in FoxO signaling in *Hydra* leads to dysregulation of antimicrobial peptide expression and that FoxO loss-of-function polyps are impaired in selection for bacteria resembling the native microbiome and more susceptible to colonization of foreign bacteria. These findings reveal a key role of FoxO signaling in the communication between host and microbiota and embed the evolutionary conserved longevity factor FoxO into the holobiont concept.

## Introduction

The aging process is considered to be the result of accumulating cellular deterioration in an individual organism over time. It can be affected by the combined influence of genetic, epigenetic, and environmental factors (López-Otín et al., 2013; Dato et al., 2017). In order to extend life and health span, one major aim of medical research is to counteract stem cell exhaustion, tissue senescence, and decline in organic and cellular functionality (Kenyon, 2010; López-Otín et al., 2013). To date, only two genetic factors have been consistently found to contribute to longevity: apolipoprotein E (Apo-E) and forkhead-box protein O3 (FoxO3) (Schächter et al., 1994; Willcox et al., 2008; Phillips, 2014; Broer et al., 2015; Morris et al., 2015). While Apo-E is considered a mortality factor and is rather specific for vertebrates, the conserved transcription factor FoxO is associated with longevity and can be found even in early branching metazoans (Bridge et al., 2010). Functional studies from various model organisms demonstrated highly conserved target sites for FoxO as well as its role in life-span extension by contributing to cell survival, stem cell control, and tissue homeostasis (Kenyon et al., 1993; Hwangbo et al., 2004; Lasi et al., 2010; Monsalve and Olmos, 2011; Boehm et al., 2012; Webb et al., 2016). However, not only are genetic predispositions driving the aging process but also environmental factors including life-style-associated factors such as exercise or diet (Dato et al., 2017; Smith et al., 2017). Furthermore, there is overwhelming evidence that symbiotic microorganisms [collectively the microbiome (Lederberg and McCray, 2001)] greatly impact the host’s development as well as tissue maintenance and therefore may also be key players in the aging process. Since all organisms are associated with a complex community of commensal bacteria, archaea, and viruses, they are considered as metaorganisms or holobionts (Eckburg, 2005; Zilber-Rosenberg and Rosenberg, 2008; Bosch and McFall-Ngai, 2011; Grice and Segre, 2011). Symbiotic bacteria can provide numerous beneficial functions to the host including support of nutritional uptake (Sandström and Moran, 1999; Shigenobu et al., 2000; Ley et al., 2005, 2006; Warnecke et al., 2007; Keeney and Finlay, 2011), detoxification of harmful substances (Chaucheyras-Durand et al., 2010), or protection from pathogenic infections (Fraune et al., 2015). Recent research also underlines the importance of bacterial colonization on host behavior (Sharon et al., 2011; Murillo-Rincon et al., 2017; Vuong et al., 2017) as well as fundamental developmental processes and immune system maturation (Rakoff-Nahoum et al., 2004; Mazmanian et al., 2005; Bates et al., 2006; Cheesman et al., 2011; Hill et al., 2016). Therefore, an organism’s health relies on stable microbial colonization (Goodman et al., 2009; Blottière et al., 2013; Khosravi and Mazmanian, 2013; Fujimura and Lynch, 2015). Disruption of the commensal bacterial community by antibiotics allows pathogens a post-treatment invasion of the host tissue (Pavia et al., 1990; Kelly et al., 1994; Pepin et al., 2005). The ability of a bacterial community to resist or compensate these incisive events and return to the stable state (resilience) is tightly connected to the onset of diseases, and therefore has become a prominent topic in microbiome research (Greenhalgh et al., 2016; Sommer et al., 2017). Assembly of the species-specific microbiome of every living organism is strictly dependent on selective mechanisms provided by the host. Such mechanisms include antimicrobial peptides (AMPs) which are important effectors of the innate immune system of all animals and also may contribute to host survival by selecting for beneficial commensal bacteria (Franzenburg et al., 2013; Fraune et al., 2015). Interestingly, as animals, including humans, age the composition of the microbiome changes and microbes may even affect the rate of aging itself (Yatsunenko et al., 2012; Heintz and Mair, 2014). However, by which processes environmental factors such as the microbiome interact with the genetic machinery controlling aging remains largely unknown.

The cnidarian *Hydra* is considered non-senescent (Schaible and Sussman, 2013) and harbors a relatively low diversity of symbiotic bacterial colonizers (Fraune and Bosch, 2007). The conserved transcription factor FoxO is a major player in stem cell differentiation and thereby is directly linked to *Hydra*’s immortality (Boehm et al., 2012, 2013). Previous approaches in *Hydra* could show that loss of tissue homeostasis or targeted knockdown of AMPs lead to changes in the symbiotic bacterial community (Fraune et al., 2009; Franzenburg et al., 2013). Moreover, certain bacterial taxa of the naturally occurring microbiome community contribute to protection against fungal infections (Fraune et al., 2015). While FoxO has been shown previously to mediate the expression of AMPs in *Hydra* and other model organisms (Evans et al., 2008; Becker et al., 2010; Boehm et al., 2012; Seiler et al., 2013; Fink et al., 2016; Mortzfeld and Bosch, 2017), the contribution of the conserved stem cell factor to microbial colonization has not yet been reported. Here, we utilized FoxO-deficient animals to directly test whether FoxO controls bacterial colonization and therefore serves as an intracellular hub protein integrating two functions that are crucially involved in aging and health in metazoans: tissue maintenance and control of metaorganism homeostasis.

## Materials and Methods

### Animal Culture

Experiments were carried out with *Hydra vulgaris* (strain AEP) (Martin et al., 1997). All lines were continuously cultured at 18°C in *Hydra* medium (HM; 0.28 mM CaCl_2_, 0.33 mM MgSO_4_, 0.5 mM NaHCO_3_, and 0.08 mM KCO_3_) according to the standard procedure (Lenhoff, 1970). The animals were fed three times a week with first instar larvae of *Artemia salina*.

### Transgenic Animals

The stable knockdown lines for FoxO (Boehm et al., 2012) were achieved by generating transgenic polyps expressing a FoxO hairpin construct fused to eGFP under control of an actin promoter (Supplementary Figure S1). The vectors were injected into *H. vulgaris* (AEP) embryos as previously described (Wittlieb et al., 2006). By selecting for eGFP expression, mass cultures with epithelial expression of the respective construct were generated. Clonal animals without any eGFP-expressing cells served as control for the corresponding line (Franzenburg et al., 2013).

### Transcriptome Analyses

For transcriptome sequencing transgenic lines were cocultured in shared HM with controls for at least four weeks in five independent replicates. After sampling, animals were frozen in TRIzol (*Thermo Fisher Scientific*) at -80°C until RNA extraction with the PureLink RNA Mini Kit (*Ambion*) according to the manufacturer’s protocol. Additionally, the optional on-column DNA digestion was performed. The RNA was eluted in 30 μl and checked for sufficient quality. If necessary, the RNA was purified using 1-butanol and diethyl ether (Krebs et al., 2009) and frozen at -80°C until further use. Total RNA sequencing with previous ribosomal depletion was performed for 10 libraries on the Illumina HiSeq2500 v4 platform, with 125 bp paired-end sequencing of 12 libraries per lane. This resulted in 30–40 million reads per sample after quality control. Quality and adapters were trimmed using PRINSEQ-lite 0.20.4 (RRID:SCR_005454) (Schmieder and Edwards, 2011) and Cutadapt 1.13 (RRID:SCR_011841) (Martin, 2011). Subsequently, mapping against the *H. vulgaris* (AEP) transcriptome (Hemmrich et al., 2012) was performed using Bowtie2 2.2.9 (RRID:SCR_005476) (Langmead and Salzberg, 2012). All downstream analyses were conducted in “R” (RRID:SCR_001905) (R Development Core Team, 2016). Differentially expressed (DE) contigs were identified with the package DESeq2 1.16.1 (RRID:SCR_000154) (Love et al., 2014). The RNA-Seq raw data are deposited at the Sequence Read Archive (SRA) and are available under the project ID SRP133287.

### Recolonization Experiment

Germ-free control and FoxO-deficient (FoxO^-^) polyps were generated using the previously established protocol (Franzenburg et al., 2012) and incubated for two days in sterile HM prior recolonization. Absence of bacteria was verified as previously described (Franzenburg et al., 2013). For recolonization, germ-free control and FoxO^-^ polyps were separated into well plates under sterile conditions. For every species, 13 polyps of *H. vulgaris* (AEP), *Hydra oligactis*, or *Hydra viridissima* were pooled and washed three times with sterile HM. After homogenization, the three suspensions were filled up to 1.3 ml and used for recolonization. Individual germ-free polyps of control and FoxO^-^ animals were recolonized with 100 μl in 4 ml HM each of one of the suspensions in six biological replicates. The same source community suspension was used to recolonize six replicates of control and FoxO^-^ polyps in a 1:1 ratio, meaning one homogenized polyp to one polyp to be recolonized. One adult polyp equated to the following colony forming units (CFU) on R2A media: *H. vulgaris* (AEP): 2.9 ^∗^ 10^4^; *H. oligactis*: 6.7 ^∗^ 10^4^; and *H. viridissima*: 2.3 ^∗^ 10^3^. The remaining 100 μl was frozen at -20°C for source community analysis. After 24 h of incubation, all polyps were washed with sterile HM. On day 4 of recolonization, the polyps were washed three times with sterile HM and frozen at -20°C for 16S rRNA profiling until DNA extraction.

### DNA Extraction and 16S rRNA Profiling

Genomic DNA was extracted from individual polyps with the DNeasy Blood & Tissue Kit (Qiagen) as described in the manufacturer’s protocol. Elution was performed in 50 μl. Extracted DNA was stored at -20°C until sequencing. Prior to sequencing, the variable regions 1 and 2 (V1V2) of the bacterial 16S rRNA genes were amplified according to the previously established protocol using the primers 27F and 338R (Rausch et al., 2016). For bacterial 16S rRNA profiling, paired-end sequencing of 2 × 300 bp was performed on the Illumina MiSeq platform. The 16S rRNA sequencing raw data are deposited at the SRA and are available under the project ID SRP128106. The sequence analysis was conducted using the QIIME 1.9.0 package (RRID:SCR_008249) (Caporaso et al., 2010). Paired-end reads were assembled using SeqPrep (RRID:SCR_013004). Chimeric sequences were identified with ChimeraSlayer (RRID:SCR_013283) (Haas et al., 2011) and verified manually before removal from the data set. If a putative chimera was present in at least two independent samples, the sequences were retained in the analysis.

### Analysis of Bacterial Communities

Operational taxonomic unit (OTU) picking was performed using the pick_open_reference_otus.py protocol with at least 97% identity per OTU and annotation was conducted with the UCLUST algorithm (RRID:SCR_011921) (Edgar, 2010) against the GreenGenes database v13.8 (RRID:SCR_002830) (DeSantis et al., 2006) implemented in QIIME. OTUs with <50 reads were removed from the data set to avoid false-positive OTUs that may originate from sequencing errors (Faith et al., 2013). The number of reads was normalized to the lowest number of reads in the dataset. This was 22,000 reads for analyses including recolonizations with bacteria from *H. vulgaris* (AEP) and *H. oligactis*. For analyses including data from the *H. viridissima* source community the number of reads was normalized to 3,200 since reads assigned to chloroplasts of the algal endosymbiont were removed *in silico*. Alpha diversity was calculated using the Chao1 metric implemented in QIIME using ten replicates of rarefication per sample. Beta diversity was depicted in a PCoA by 100 jackknifed replicates using binary Pearson distances (BPDs). Bacterial groups specifically associated with control or FoxO^-^ polyps were identified by LEfSe (RRID:SCR_014609) (Segata et al., 2011). LEfSe couples robust tests for measuring statistical significance (Kruskal–Wallis test) with quantitative tests for biological consistency (Wilcoxon signed-rank sum test). The differentially abundant and biologically relevant bacterial groups are ranked by effect size after undergoing linear discriminant analysis. All *p*-values were corrected for multiple hypotheses testing using Benjamini and Hochberg’s false-discovery rate correction (*q*-value). A *q*-value of 0.25, an effect size threshold of 3.0 (on a log_10_ scale), and a mean abundance of at least 0.1% in one of the treatments were used for all bacterial groups discussed in this study.

### Statistics

Statistical analyses were performed using two-tailed Student’s *t*-test or Mann–Whitney *U*-test if applicable. If multiple testing was performed, *p*-values were adjusted using the Benjamini–Hochberg correction (Benjamini and Hochberg, 1995).

## Results

### FoxO Controls Multiple Families of AMPs

In order to investigate the effect of FoxO on innate immunity and metaorganism homeostasis in a functional approach, we generated transgenic animals carrying a shRNA construct against the single *Hydra foxO* gene (contig 8319) to disrupt FoxO-dependent signaling (Supplementary Figure S1). We targeted the epithelial cell lines to investigate their influence on innate immunity functions. In a previous study, we could show that whole AMP families can be affected by dysregulation of FoxO (Boehm et al., 2012); however, to specifically evaluate the expression of individual AMP contigs, we performed a comparative transcriptome approach using FoxO-deficient (FoxO^-^) animals for the epithelial tissue.

**FIGURE 1:**
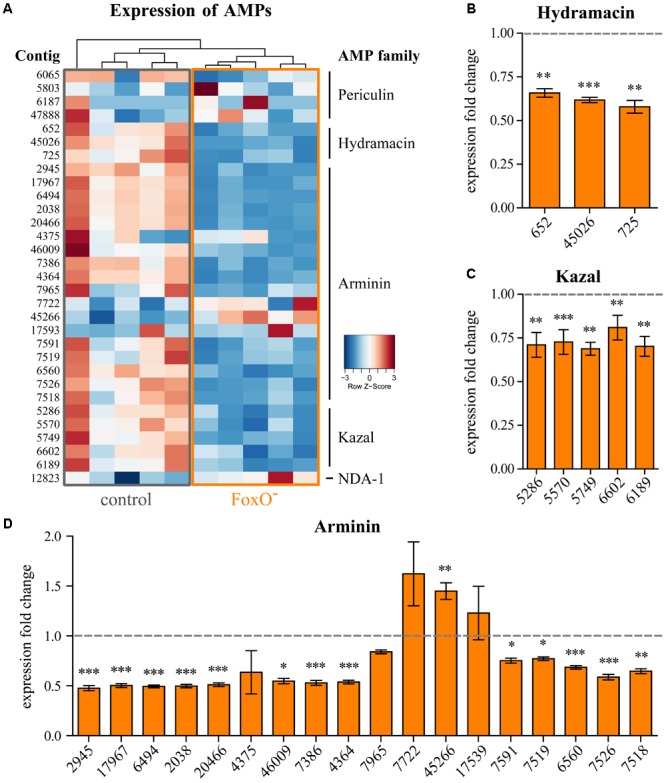
Expression of individual AMP contigs. **(A)** Heatmap representing the relative expression level of the contigs of the AMP families Periculin, Hydramacin, Arminin, Kazal, NDA-1 for control, and epithelial FoxO^-^ animals. Samples were clustered hierarchically. Expression values were log_2_-transformed and median-centered by transcript. **(B–D)** Expression fold change of individual AMP contigs belonging to the families Hydramacin, Kazal, or Arminin compared to the control samples. Error bars represent standard error of the mean (SEM). Statistical tests were performed on normalized read counts shown in Supplementary Figure S4. *n* = 5, ^∗^*p* ≤ 0.05, ^∗∗^*p* ≤ 0.01, ^∗∗∗^*p* ≤ 0.001.

For comparative expression analysis, we sequenced ten total RNA libraries with five biological replicates of control and FoxO^-^ animals, resulting in 30–40 million reads per sample after quality control. In order to distinguish even closely related contigs within one AMP family, we utilized the uniquely mapped hits to calculate DE contigs. Principal component analysis of the transcriptomes revealed significant changes in gene expression by disruption of FoxO signaling. Clustering of control and FoxO^-^ samples explained >50% of variance in the dataset (Supplementary Figure S2), suggesting a strong influence of FoxO-dependent signaling on target gene expression. **Figure 1A** displays a heatmap of all previously described AMP families in *Hydra* with corresponding contig numbers for control and FoxO^-^ polyps (Augustin et al., 2009, 2017; Jung et al., 2009; Fraune et al., 2010; Franzenburg et al., 2013). FoxO deficiency has a strong impact on the innate immune system and the expression of AMPs. The relative expression of all ten samples reveals the members of the AMP families Hydramacin, Arminin, and Kazal to be downregulated in FoxO^-^ polyps. No effect could be observed for the Periculins and NDA-1 which are known to be predominantly expressed in the interstitial stem cell lineage (Fraune et al., 2010; Augustin et al., 2017) and are not affected by disruption of FoxO-dependent signaling in the epithelial tissue (Supplementary Figures S3, S4D,E). Interestingly, all three contigs coding for Hydramacins (Jung et al., 2009) are downregulated by 34–42% upon FoxO deficiency (**Figure 1B**), while the Kazal protease inhibitors (Augustin et al., 2009) show dysregulation of 23–31% in all five contigs (**Figure 1C**). The largest family of AMPs, the Arminins (Franzenburg et al., 2013), is also the most affected one (**Figure 1D**). Most contigs respond with significant downregulation up to 52% in FoxO^-^. However, for four individual contigs of the Arminin AMP family (4375, 7965, 7722, and 17539), we could not find significant changes in expression. Remarkably, evaluating normalized read counts (Supplementary Figure S4), contig 7965 in the Arminin family shows the highest expression level but the weakest response to FoxO deficiency. Moreover, contig 45266 is the only example of all investigated AMP contigs responding with significant upregulation in FoxO^-^ polyps (**Figure 1D**).

Downregulation of the *foxO* transcript in the epithelial tissue leads to an overall downregulation of epithelially expressed AMPs. The extent of regulation for all AMP contigs shows strong similarities within the families and seems to be independent of the expression level (Supplementary Figure S4). This suggests no individual targeting of peptides but a rather common activating mechanism of AMP expression by the FoxO transcription factor.

### FoxO Affects Microbiome Resilience

**FIGURE 2:**
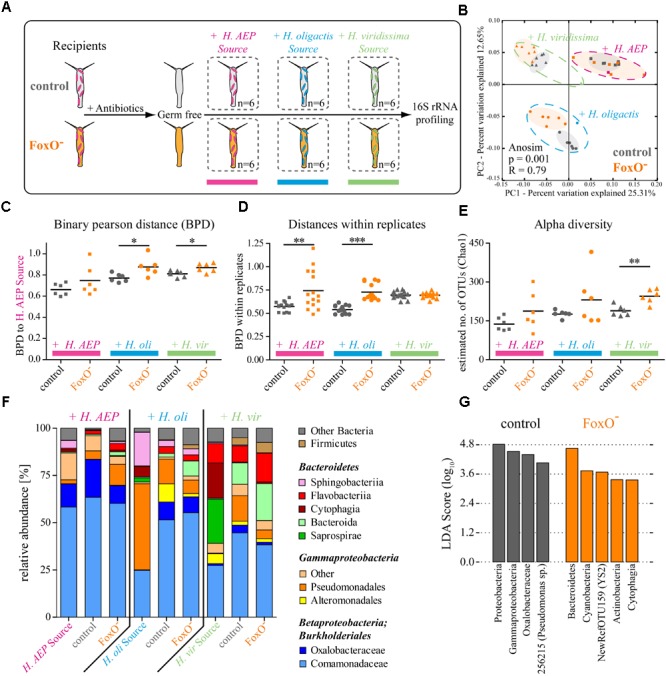
FoxO controls metaorganism homeostasis and microbiome resilience. **(A)** Schematic representation of the experimental design. Germ-free polyps of control and FoxO^-^ (*H. vulgaris* (AEP)) were recolonized with bacterial sources either from the same species (*H.* AEP) or species-specific bacteria from *H. oligactis* or *H. viridissima* for 4 days. **(B)** Analysis of bacterial communities associated with control or FoxO^-^ polyps after recolonization with different bacterial sources using principal coordinate analysis (PCoA) of the binary Pearson distance (BPD) matrix. Note the distinct clusters for the different bacterial backgrounds as well as the differences in recolonization outcome for control and FoxO^-^ animals. Ellipses were added manually. Dashed ellipses group samples for source communities and ANOSIM analysis. The percent variation explained by the PCoA is indicated on the axes. **(C)** BPDs of control and FoxO^-^ polyps recolonized with different bacterial source communities to the *H. vulgaris* (AEP) source community. **(D)** Within treatment BPDs of control and FoxO^-^ animals representing the diversity variability between the biological replicates. **(E)** Choa1 estimated alpha diversities for control and FoxO^-^ after recolonization. **(F)** Bar charts representing the microbiota of source communities and recipient polyps with mean relative abundances of bacterial classes. **(G)** Linear discriminant analysis of effect sizes (LEfSe) results for recolonization of control and FoxO^-^ polyps over all bacterial backgrounds, indicating bacterial groups specific for one of the conditions. ^∗^*p* ≤ 0.05, ^∗∗^*p* ≤ 0.01, ^∗∗∗^*p* ≤ 0.001.

Our observations demonstrate that *foxO* expression has a broad positive impact on AMP expression (**Figure 1**). Assuming that AMPs affect microbial colonization (Franzenburg et al., 2013), we next hypothesized that FoxO is thereby also able to affect bacterial colonization and microbiome resilience. In order to functionally test this, we performed recolonization experiments offering the native microbiome or bacterial sources from *Hydra* species that are known to harbor substantially different bacterial communities (Franzenburg et al., 2013; **Figure 2A**). We generated germ-free control and FoxO^-^ polyps and separated them into single wells under sterile conditions. Independently, we provided the bacteria-free animals with source communities for 24 h from either their own species *H. vulgaris* (AEP) or from other *Hydra* species, *H. oligactis* or *H. viridissima*. After four days of recolonization, single polyps of the six biological replicates were subjected to 16S rRNA profiling of the bacterial colonizers *via* sequencing on the Illumina MiSeq platform (**Figure 2A**).

Principle coordinate analysis (PCoA) on a binary Pearson distance (BPD) matrix of the composition data displayed distinct clustering for the different bacterial backgrounds, verifying the successful recolonization with the provided source communities (**Figure 2B**: pink, blue, green; Supplementary Table S1). Recolonization of control and FoxO^-^ polyps with the native bacterial community (*H*. AEP) did not result in significantly different outcomes [analysis of similarities (ANOSIM): *p* = 0.112, *R* = 0.17] (**Figure 2B** and Supplementary Table S1). However, when provided with foreign bacteria from *H. oligactis* (ANOSIM: *p* = 0.001, *R* = 0.45) or *H. viridissima* (ANOSIM: *p* = 0.001, *R* = 0.47), the recolonization resulted in distinct clusters for FoxO^-^ and control polyps (Supplementary Table S1). Moreover, quantification of BPDs to the *H. vulgaris* (AEP) source community confirms that FoxO^-^ polyps are significantly impaired in selecting bacteria resembling the native microbiome, when presented with foreign bacterial backgrounds (**Figure 2C**). Their BPDs increase significantly when recolonized with bacteria from *H. oligactis* (0.77 ± 0.04 to 0.88 ± 0.09; mean ± SD) or *H. viridissima* (0.81 ± 0.03 to 0.87 ± 0.04). Remarkably, even during recolonization with naturally occurring bacteria the resilience was drastically decreased, as the within treatment variability for the FoxO^-^ animals was significantly increased compared to the control (0.57 ± 0.05 to 0.74 ± 0.21) (**Figure 2D**). Evaluating the alpha diversity, FoxO-deficient polyps were less efficient in controlling the number of bacterial OTUs (**Figure 2E**). While FoxO^-^ polyps recolonized with bacteria from *H. viridissima* clearly showed a significant increase in OTUs (189 ± 21 to 245 ± 27) (**Figure 2E**), animals provided sources from *H. vulgaris* (AEP) (138 ± 26 to 188 ± 73; *F*-test: *p* = 0.038) or *H. oligactis* (176 ± 14 to 230 ± 102; *F*-test: *p* = 0.0005) showed significantly higher variation in alpha diversity compared to the control, suggesting a higher prevalence of stochastic events in FoxO^-^ animals.

**Figure 2F** depicts the relative bacterial abundances of the source communities as well as the control and FoxO^-^-recipient polyps. When colonized with bacteria from *H. vulgaris* (AEP), native bacteria could settle on the polyp and the bacterial communities almost resembled the diversity of the source community for control and FoxO^-^ polyps already four days after recolonization. Remarkably, when recolonized with bacteria from other *Hydra* species, control and FoxO-deficient animals select for bacterial taxa resembling their native microbiota (*H.* AEP) from the source community. Even some highly abundant members of the foreign source communities, such as *Sphingobacteriia* (*H. oli*), *Sapropirae*, or *Cytophagia* (*H. vir*), were poorly transmitted. Interestingly, bacterial orders that are barely present in the source or the native *H. vulgaris* (AEP), like *Bacteroida, Flavobacteriia, Alteromonadales*, or *Firmicutes*, were able to expand at the drawback of bacteria resembling the native microbial composition (**Figure 2F** and Supplementary Table S2).

When evaluating the differences between control and FoxO-deficient animals for the foreign bacterial backgrounds using linear discriminant analysis effect sizes (LEfSe) (Segata et al., 2011), it becomes evident that especially bacterial taxa that are rarely found in the native *H. vulgaris* (AEP) community are able to reach higher relative abundances and thereby reduce the portion of *Beta*- and *Gammaproteobacteria* (Supplementary Figure S5). Particularly, OTUs that are classified as *Bacteroida* (e.g., 348038, 267046, and 179810), *Flavobacteriia* (2708478), *Cyanobacteria* (NewRefOTU159), and *Actinobacteria* (1098473) can establish in higher abundances on FoxO^-^ animals (Supplementary Figure S5 and Supplementary Table S3). Reviewing the effects of FoxO deficiency despite the bacterial background for recolonization, higher proportions of *Bacteroidetes* (*Cytophagia*), *Cyanobacteria* (*YS2*), and *Actinobacteria* could be observed consistently on the FoxO^-^ polyps (**Figure 2G**). Due to the presence of host species-specific bacterial taxa in the different source communities, there is only little overlap of commonly affected OTUs over the whole data set. However, all the rising bacterial groups are usually absent or only found in very low abundances in the naturally occurring *H. vulgaris* (AEP) microbiota (Franzenburg et al., 2013) (Supplementary Table S3). Vice versa, control animals always showed higher abundances of known commensal bacterial classes compared to FoxO^-^ animals, belonging to *Betaproteobacteria* (*Oxalobacteraceae*) and *Gammaproteobacteria* (*Pseudomonas* sp.) (**Figure 2G**).

Taken together, our observations demonstrate that FoxO-dependent signaling not only affects the expression of AMPs, but also has the potential to mediate abundances and diversity of microbes by controlling selectivity for bacterial colonizers. FoxO deficiency significantly increases variability of recolonization outcome with a native bacterial *H. vulgaris* (AEP) source community and drastically reduces resilience when provided bacteria from other *Hydra* species. Especially bacterial taxa that are rarely found in the native community can expand at the drawback of bacteria resembling the naturally occurring microbiota.

## Discussion

**FIGURE 3:**
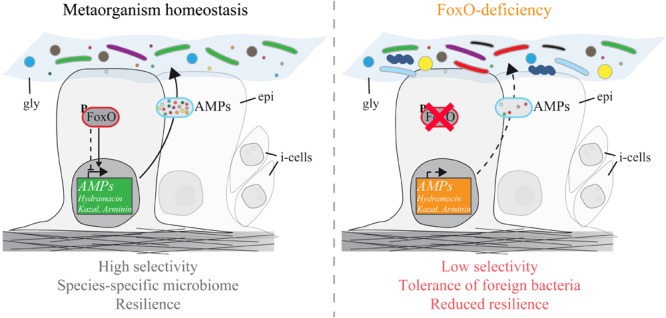
Schematic representation showing the effect of FoxO on AMP expression and metaorganism homeostasis. (Left) In a state of intact FoxO signaling and metaorganism homeostasis, FoxO acts as an activator on the expression of AMPs of the families Hydramacin, Kazal, and Arminin in the epithelial tissue (epi). This results in a unique cocktail of AMPs in the glycocalyx (gly) of the animals, shaping the microbiome in a species-specific manner. (Right) FoxO deficiency reduces the overall expression of AMPs. Decreased levels of AMPs reduce the host selection over symbiotic bacteria colonizing the glycocalyx. Due to a higher tolerance an increased diversity of non-species-specific bacteria can settle. This demonstrates impairment in microbiome resilience and metaorganism homeostasis caused by FoxO deficiency. i-cells: interstitial stem cells.

The non-senescent *Hydra* holobiont is a valuable tool to study conserved mechanisms of host–microbe interactions in functional analyses (Deines and Bosch, 2016; Schröder and Bosch, 2016). By exploring epithelial FoxO loss-of-function mutants, we made two important discoveries. First, deficiency in FoxO signaling leads to dysregulation of multiple AMP families (**Figure 1**). Most genes encoding epithelially expressed AMP families including Hydramacin, Kazal, and Arminin respond with downregulation to FoxO deficiency. Only one gene (contig 45266) was found to be upregulated in FoxO-deficient animals, suggesting a mainly activating function of FoxO signaling on AMP expression and innate immunity. Second, FoxO loss-of-function polyps were more susceptible to colonization of foreign bacteria and impaired in selection for bacteria resembling the native microbiome (**Figure 2**). Therefore, FoxO-induced decrease in AMP expression is correlating with differences in microbial colonization and highlights the inhibitory action of AMPs against non-commensal bacteria. In a state of intact FoxO signaling, secretion of numerous AMP families provides a highly selective milieu and shapes the microbial composition in a species-specific manner (**Figure 3**). FoxO deficiency reduces the expression of AMPs, which results in a decreased selection pressure on colonizing taxa and in establishment of higher abundances of foreign bacteria in the community. Consequently, especially during the process of colonization, reduced expression of FoxO compromises the resilience of the microbiome (**Figure 3**).

The last decades of molecular research and observational GWAS studies have provided increasing evidence that FoxO is able to delay the aging process and thereby extend the health period and life span all across the animal kingdom (Boehm et al., 2013; Martins et al., 2016). Its involvement in oxidative stress responses, DNA damage repair, apoptosis, and cell cycle control makes it a key regulator in tissue maintenance (Webb and Brunet, 2014; Morris et al., 2015; Martins et al., 2016). Scientists are only recently recognizing the important influence the microbiome may have on the aging process and many end-of-life diseases (Kundu et al., 2017). The aging process affects the structure of the human gut microbiota which leads to a decrease in species diversity resulting in a higher risk of pathogenic infections, age-related constipation, and low-level chronic inflammation (known as “inflammaging”) in elderly populations (Biagi et al., 2012; Candela et al., 2014; Keller and Surawicz, 2014; Kundu et al., 2017). Interestingly, we observed an increase in microbial diversity in FoxO^-^ animals. We would explain this by the early state of recolonization that we present in this study. The naturally occurring diversity was nearly completely restored for control polyps after 4 days of recolonization (**Figure 2F**, *H*. AEP), while FoxO^-^ animals were less efficient in controlling the bacterial taxa provided with the source communities (**Figures 2C–E**). Since the host factor controlling the bacterial colonization is reduced in FoxO^-^ animals, long-term effects may be shaped by a higher degree of bacteria–bacteria interactions resulting in overgrowth of few dominant taxa. This would reduce the overall diversity as observed in other studies. Consequently, the observed reduction in diversity during the aging process in other models may result from the decreasing control of dominant bacterial colonizers expanding in the community, while rare members are repressed. Since the structure of the bacterial community can be protective against pathogens, can shape the nutrient landscape, and may affect the inflammation status, evidence is accumulating that it has a direct influence on senescence in humans (Heintz and Mair, 2014) or even is responsible for the aging process itself (Blaser and Webb, 2014). A study focused on the short-lived African killifish for example revealed that transplantation of a bacterial community from a young to an old individual is able to expand host life span (Smith et al., 2017). Similarly, in the nematode *Caenorhabditis elegans*, non-essential bacterial compounds are able to regulate mitochondrial dynamics and unfolded protein response and thereby directly affect host life span and health span (Han et al., 2017).

Taken together, observations in different organisms indicate that, in contrast to the essentially static genome, the microbiome is rather dynamic throughout life history. Our observations in non-senescent *Hydra* add support to the view that (i) there is a need to consider the holobiotic nature of an organism when thinking about longevity, that (ii) the microbial environment matters in the context of senescence and contributes to complex processes such as aging; and that (iii) the hub regulator FoxO presents a direct link between age-related processes and microbial colonization.

## Ethics Statement

Ethical restrictions do not apply to cnidarian model organisms such as *Hydra*.

## Author Contributions

BM, SF, AK, and TB designed the experiments. BM performed the experiments. BM, JT, SF, AK, and TB analyzed the data. BM and TB wrote the paper.

## Conflict of Interest Statement

The authors declare that the research was conducted in the absence of any commercial or financial relationships that could be construed as a potential conflict of interest.
