# Supplementary figures and images for: Stem Cell Transcription Factor FoxO Controls Microbiome Resilience in Hydra
Source: Front Microbiol. 2018 Apr 3;9:629. doi: 10.3389/fmicb.2018.00629 (PMC5891625; doi:10.3389/fmicb.2018.00629)

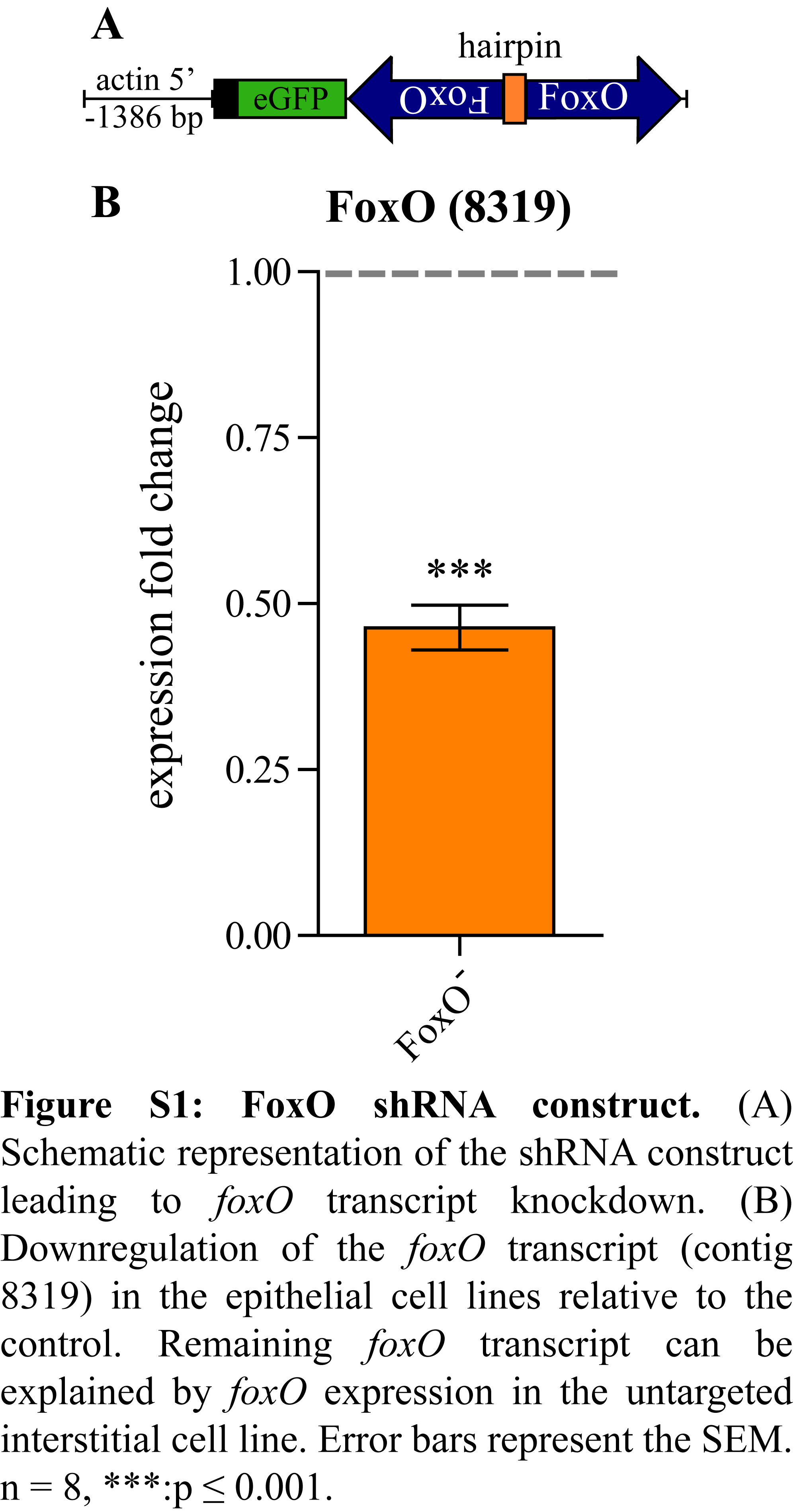

Supplement: Supplementary file 4 [file Image_1.PNG]

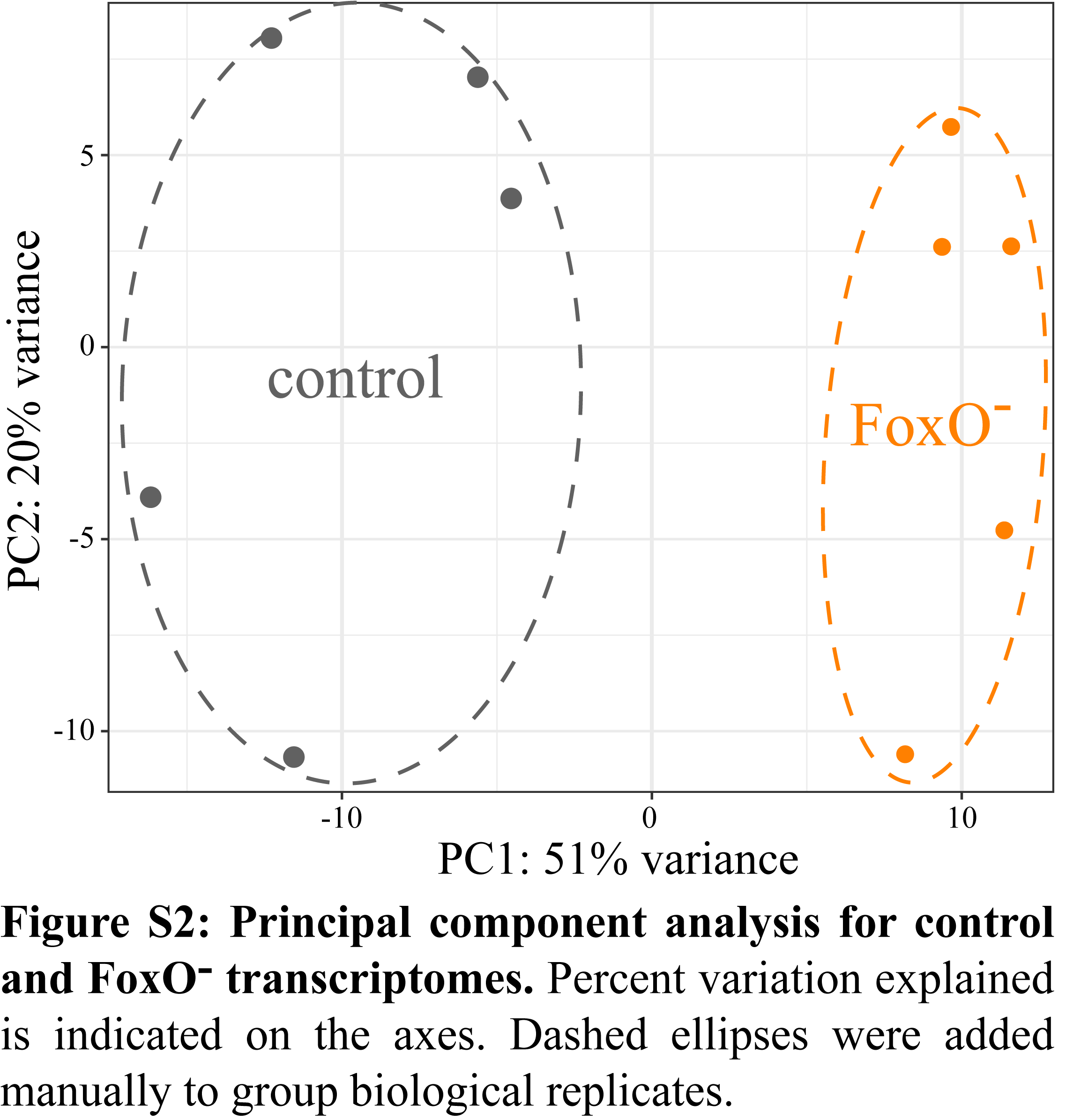

Supplement: Supplementary file 5 [file Image_2.PNG]

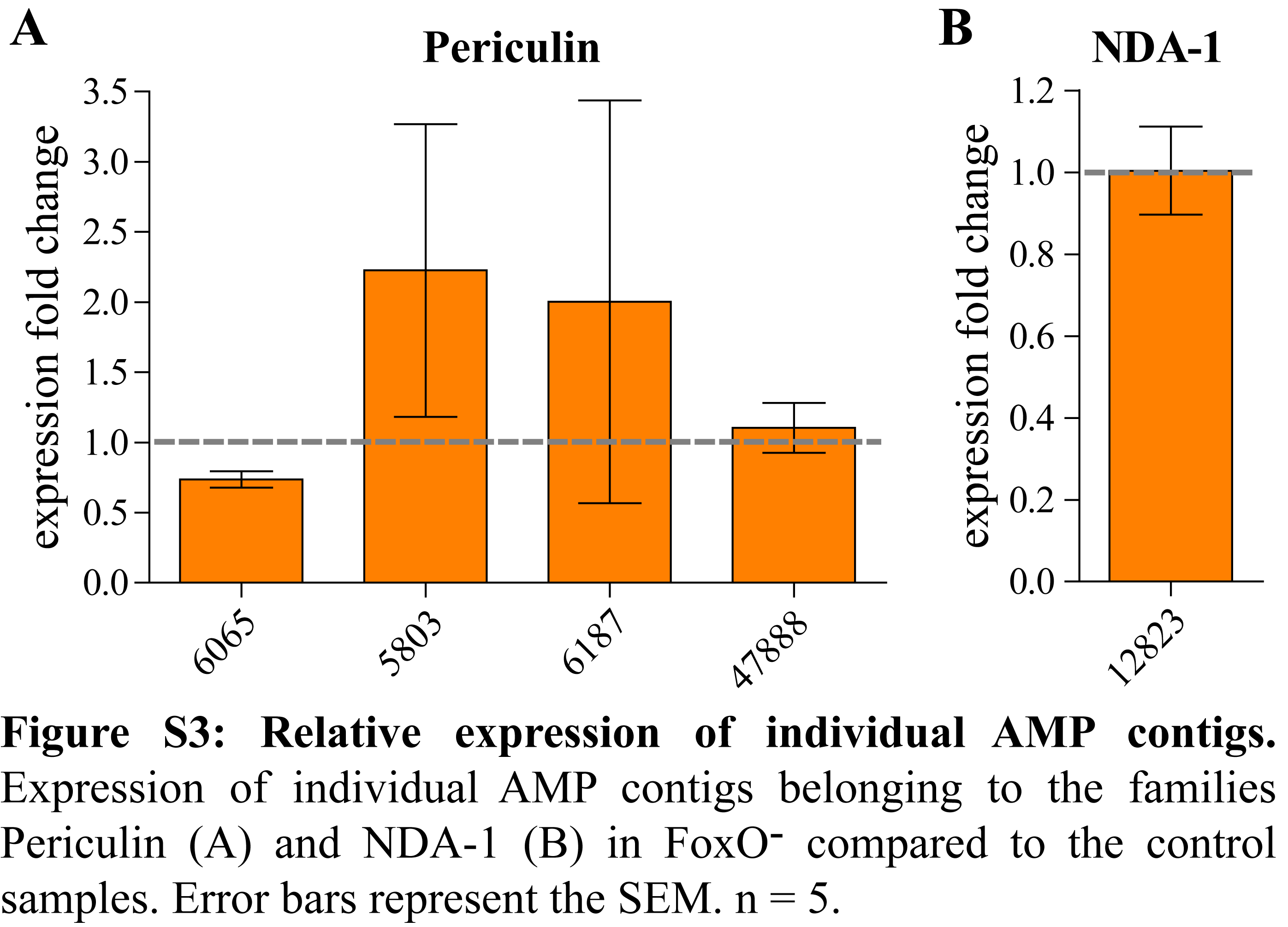

Supplement: Supplementary file 6 [file Image_3.PNG]

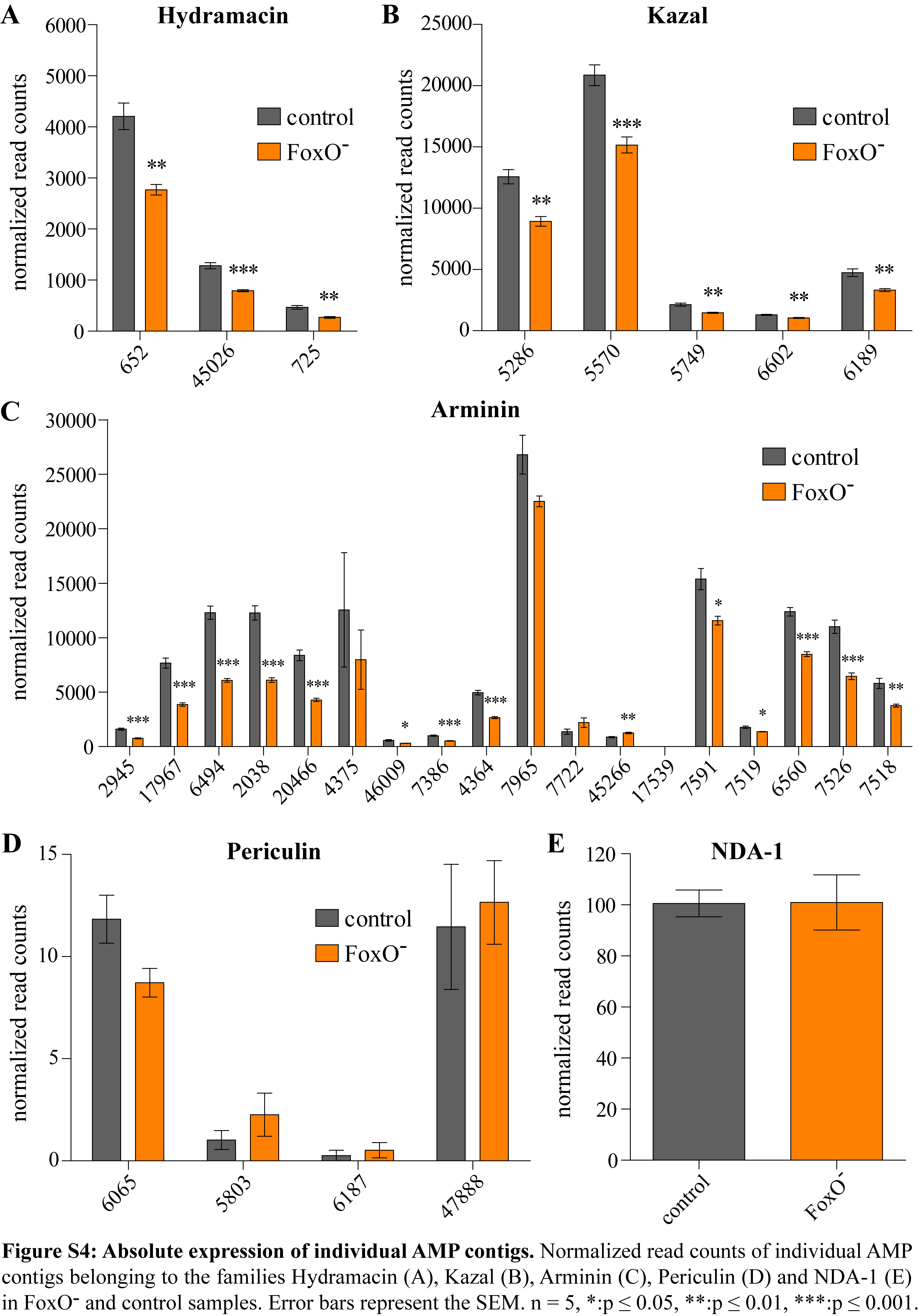

Supplement: Supplementary file 7 [file Image_4.PNG]

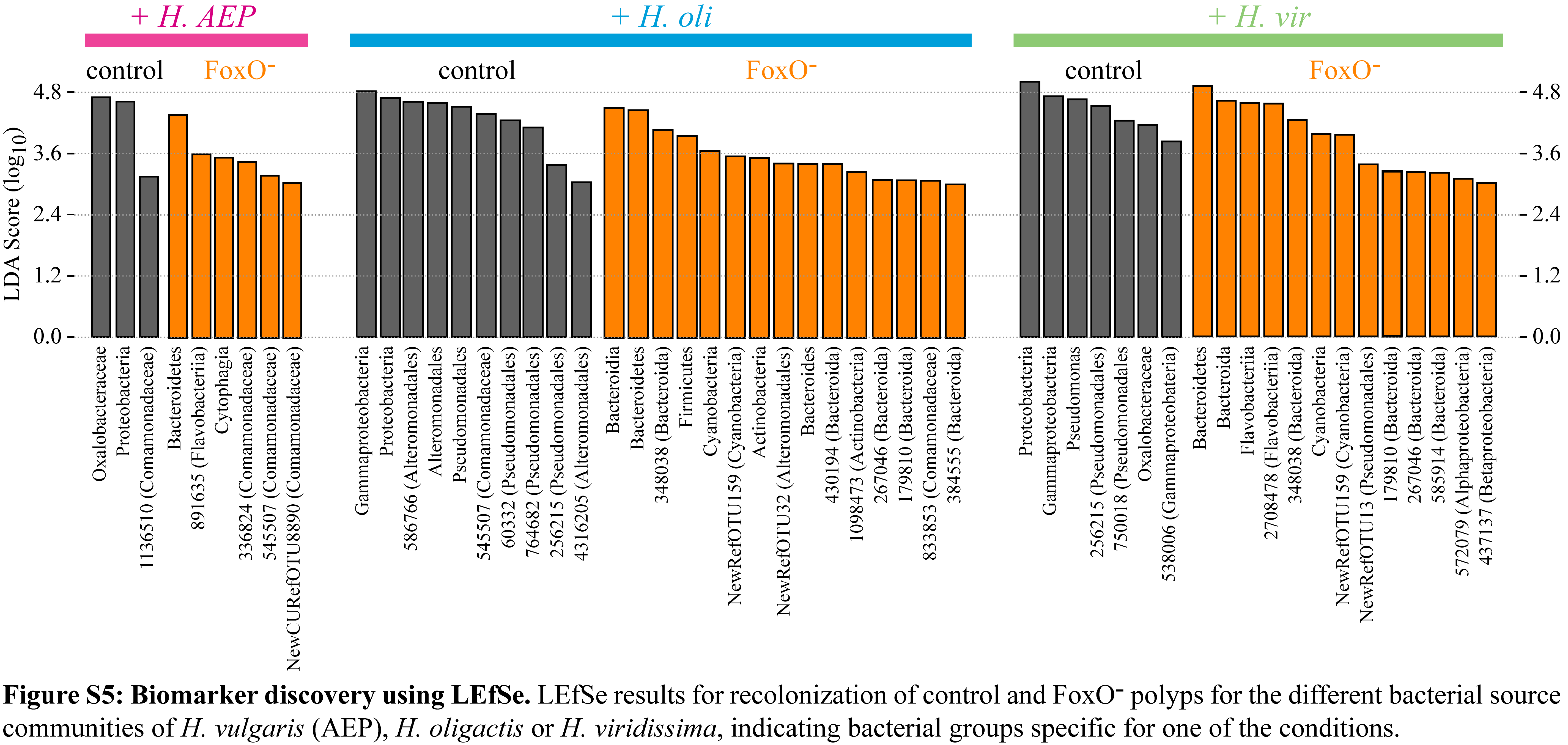

Supplement: Supplementary file 8 [file Image_5.PNG]
